# Which Is More Influential in University Teachers: Work Interfering with Family or Family Interfering with Work? A Network Analysis

**DOI:** 10.3390/bs16030322

**Published:** 2026-02-26

**Authors:** Weiwei Zhu, Ren Gan, Di Liu, Xinglin Luo

**Affiliations:** School of Psychology, Qufu Normal University, Jining 273165, China; ganren@qfnu.edu.cn (R.G.); liudi@qfnu.edu.cn (D.L.); luoxinglin@qfnu.edu.cn (X.L.)

**Keywords:** work–family conflict, job burnout, work engagement, work connectivity behaviors after-hours, university teachers, network analysis

## Abstract

As university teachers face increasing work pressure and family responsibilities, Work–Family Conflict (WFC) has become a significant factor affecting teachers’ psychological health. Based on the Conservation of Resources Theory and network analysis methods, this exploratory study investigates the systematic impact of WFC on teacher job burnout, work engagement, and work connectivity behaviors after-hours. Through data analysis of a sample of 409 university teachers, a partial correlation network model was constructed, revealing patterns, central nodes, and conditional associations between variables that describe how WFC affects the psychological system of teachers. The results show that “Work Interfering with Family (WIF)” holds the highest centrality and bridging effect in the network, acting as a key node connecting multiple dimensions of job burnout, work engagement, and work connectivity behaviors after-hours. Specifically, WIF is directly associated with emotional exhaustion and indirectly weakens work engagement through the mediation of reduced professional efficacy. Additionally, work connectivity behaviors after-hours (WCBA) have a close and significant influence on WIF. This study highlights the pivotal role of WFC in relation to teachers’ job burnout, work engagement, and WCBA, providing an exploratory perspective and precise intervention guidelines for intervention strategies.

## 1. Introduction

With the intensification of global competition and the continuous evolution of work patterns, university teachers are facing increasing pressures from both work and family. Work–family conflict is defined as role conflict that arises when the demands of work and family roles are incompatible (Greenhaus & Beutell, 1985). This conflict is a critical factor shaping individuals’ psychological health and career trajectories, as it directly impacts core aspects of professional functioning and personal well-being. WFC consists of two main dimensions: Work Interfering with Family (WIF) and Family Interfering with Work (FIW) (Casper et al., 2007). The former refers to work responsibilities hindering the fulfillment of family roles, while the latter refers to family responsibilities interfering with work duties. For university teachers, this conflict is particularly salient due to the unique demands of their profession: the interconnected roles of teaching, research, and social services often create intense competition for limited resources such as time and energy, especially amid high-intensity, autonomous, and time-consuming tasks (Su & Jiang, 2023). Moreover, the widespread use of digital communication technologies and flexible work mechanisms has blurred the boundaries between work and family, making WFC a central issue in the psychological health and career development of university teachers (Geraldes et al., 2025). For teachers, mental health is closely intertwined with job satisfaction, self-efficacy, and burnout, forming a complex interactive system (Capone & Petrillo, 2020), which further highlights the necessity of exploring the impact of WFC on psychological states (job burnout and work engagement) and behavioral work outcome (work connectivity behavior after hours). In previous studies, WFC has been regarded as an important factor affecting individuals’ mental health. Existing research has shown that kindergarten teachers exhibit different impact patterns across different dimensions of WFC—for example, the degree of WIF is higher than that of FIW, and these conflicts are significantly correlated with teachers’ job burnout (Hetrick et al., 2024). Among primary and secondary school teachers, studies have also found that scores on the WIF dimension are significantly higher than those on FIW, and such conflicts are significantly positively correlated with all dimensions of job burnout, with the most prominent impact on emotional exhaustion. Additionally, self-determination motivation plays a mediating role in the relationship—fully mediating the relationship between WIF and job burnout, and partially mediating the relationship between FIW and job burnout (M. J. Li et al., 2015). WFC is prevalent among university faculty and often exhibits notable gender differences. Previous research has shown that female teachers report significantly higher levels of both work-to-family conflict and family-to-work conflict than their male counterparts, suggesting that women experience greater dual pressures between professional and family roles (Mody et al., 2022). However, the literature has not reached a consistent conclusion. Some empirical studies in the education sector indicate that there are no significant gender differences in the overall level of WFC among teachers (Johnston et al., 2023). Thus, whether stable gender disparities exist in the experience and magnitude of WFC remains a debated issue in the literature (Koura et al., 2020). As a typical high-investment and high-pressure occupational group, university faculty members continuously juggle teaching, research, service responsibilities, and family obligations, making WFC an important factor affecting their occupational health and job performance. In this context, Research has examined the mechanism through which family-to-work conflict (FWC) influences innovative work behavior using survey data from 916 Chinese university teachers. The results showed that gender significantly moderates the relationships between family-to-work conflict and challenge stress and between family-to-work conflict and hindrance stress (Bao et al., 2025). Gender plays a non-negligible role in research on WFC.

This study is based on the Conservation of Resources Theory (Hobfoll, 1989, 2001), which posits that individuals are driven by the fundamental need to acquire, maintain, and protect resources such as time, energy, emotions, or cognitive abilities. When these resources are threatened, depleted, or not adequately replenished, psychological stress and negative adaptive responses may occur. WFC is considered a typical source of resource threat, as it not only depletes individuals’ time resources but also disrupts recovery processes, leading to a range of negative impacts on work-related psychological states. In terms of work-related psychological states, job burnout and work engagement are two important dimensions that are closely intertwined with WFC and warrant focused investigation.

Job burnout refers to the symptoms of emotional exhaustion, cynicism, and reduced professional efficacy exhibited by individuals under prolonged stress (Maslach et al., 2001; Bakker & De Vries, 2021), with emotional exhaustion being considered the core feature (Anjum et al., 2022). From the perspective of COR theory, continuous WFC depletes teachers’ cognitive and emotional resources, making them more vulnerable to job burnout (Wu et al., 2018; Song et al., 2025). A meta-analysis integrating 8800 teacher samples found that WFC is highly positively correlated with job burnout among primary and secondary school teachers (r = 0.531), and this association is not moderated by factors such as educational level or teaching experience (Zhao, 2023). Furthermore, job burnout is not only a consequence of WFC but may also exacerbate conflicts through the feedback effect of resource depletion (Rubio et al., 2015; Raja et al., 2018). Additionally, teacher confidence in professional training is closely predicted by work engagement and job burnout (Fiorilli et al., 2020), highlighting the far-reaching chain effects of WFC on professional development.

In contrast, work engagement is a work-related psychological state, characterized by vigor, dedication, and absorption that individuals experience in their work (Schaufeli et al., 2002; Usset et al., 2024). Though once viewed as the opposite of job burnout, research confirms the two are structurally independent and driven by distinct mechanisms (Balogun & Afolabi, 2019). Teachers’ work engagement depends on both psychological and situational resources. WFC, as a source of resource depletion, has been shown to negatively impact teachers’ work engagement. For instance, among rural preschool teachers, WFC negatively affects work engagement, with psychological capital acting as a moderator (Lyu & Fan, 2022). Moreover, factors such as social pressure and work intensity also influence teachers’ work engagement and well-being, particularly among those facing high work pressure and intensity (Shao et al., 2025). High-intensity work engagement, when not effectively managed, may negatively affect employees’ family life quality (Listau et al., 2017). For example, the length of work hours and workload directly contribute to work–life conflict, impacting health (Skinner & Pocock, 2008). Research on primary and secondary school teachers also shows a significant positive correlation between work engagement and life satisfaction (Yuan & Wang, 2025), emphasizing the importance of reasonable work time management. Empirical studies have also found that the impact of WFC on work engagement varies across different industries (Agarwal et al., 2025; Purwayoga et al., 2019), indicating the need to reassess its impact model within the specific context of university teachers.

The widespread use of communication and computing devices, along with the continuous development of information transmission technologies, has made it increasingly common for individuals to engage with work-related matters during non-work hours and outside of official workspaces (Wang & Li, 2023). This phenomenon, known as Work Connectivity Behavior After-hours (WCBA), refers to teachers continuing to handle work-related tasks, respond to work communications, or engage in work discussions using communication tools after working hours (Ma et al., 2016). Although this behavior reflects a sense of professional responsibility, it extends the influence of work-related stress into family life and personal time, intensifying the resource conflict between work and family roles, which may have a significant impact on teachers’ psychological well-being and professional functioning. With the ongoing expansion of the teaching workforce and increasing challenges in the education sector, the professional pressures faced by teachers have escalated (Tian et al., 2024), making the study of WFC and its impact mechanisms especially urgent. In addition, WCBA, as an extension variable at the behavioral level, has long been overlooked. This behavior not only reflects WFC but may also act as a mechanism for moderating or amplifying professional stress, having a sustained impact on individual psychological states. If WCBA is not effectively managed over the long term, teachers may find it difficult to truly disengage from work, hindering the recovery of their psychological resources and even leading to burnout or reduced work motivation.

However, existing studies on work–family conflict (WFC) among teachers have largely relied on mediation or moderation models that examine linear relations between isolated variables. Such approaches are limited in their ability to capture the complex pattern of mutual associations among WFC, job burnout, work engagement, and WCBA, which may co-occur as interconnected psychological states and behavioral outcomes within teachers’ work experiences. Rather than assuming directional effects, it is necessary to understand how these components are structurally organized and related to one another within a broader system. To address this limitation, the present study adopts Psychological Network Analysis, an analytical framework that conceptualizes variables as mutually connected components of a system. By estimating edge weights, centrality, and predictability, this method identifies the relative importance and connectivity of each component and clarifies how WFC is positioned among teachers’ work-related psychological states and behavioral outcomes (Epskamp & Fried, 2018). Compared with traditional single-path models, network analysis provides a structural perspective that expands current knowledge of teacher WFC by revealing patterns of association rather than assuming causal pathways.

In summary, this study focuses on university teachers and uses Psychological Network Analysis to examine the network structure linking WFC, job burnout, work engagement, and WCBA at the dimensional level. The aim is to clarify the interrelationships among these components and to determine the relative centrality of WFC and its dimensions within the network. Specifically, this study addresses the following research questions:

1. What is the network structure linking work–family conflict (WFC) with university teachers’ work-related psychological states (job burnout and work engagement) and behavioral work outcome (work connectivity behavior after hours)?
2. Within this network, which components are most central in the associations between WFC and teachers’ work-related psychological states and behavioral work outcome?
3. Between the two dimensions of WFC (WIF and FIW), which occupies a more central position in the network connecting WFC with job burnout, work engagement, and after-hours work connectivity behavior?

## 2. Method

### 2.1. Participants and Procedure

This study used a group sampling method, selecting teachers from several universities in a province in East China. A total of 490 questionnaires were mailed out, and 409 valid responses were collected, yielding a response rate of 83.5%. Basic demographic information about the participants, such as gender, age, and marital status, is shown in Table 1.

### 2.2. Measures

Work–Family Conflict: WFC was measured using the scale developed by Carlson et al. (2000), which includes two subscales: WIF and FIW. Each subscale consists of three dimensions: behavioral conflict, time conflict, and stress conflict, with a total of 18 items. A 5-point Likert scale was used, where 1 represents “strongly disagree” and 5 represents “strongly agree,” with higher scores indicating greater levels of WFC. The Cronbach’s alpha for the total scale was 0.93, and the Cronbach’s alpha for WIF and FIW subscales were 0.92 and 0.91, respectively. Reliability estimates for all scales are in Table 2.

Job Burnout: The Chinese version of the Maslach Burnout Inventory–General Survey (MBI–GS) revised by C. P. Li and Shi (2003) was used to investigate the job burnout status of the research objects. The scale consists of 15 items, divided into three dimensions: “Emotional Exhaustion” (5 items), “Cynicism” (4 items), and “Reduced Professional Efficacy” (6 items). Each item was scored using a Likert 7-point scale, ranging from 0 (“never”) to 6 (“very frequently”). The score of each dimension was the average score of all items in that dimension; higher scores on the Emotional Exhaustion and Cynicism dimensions indicated more severe job burnout, while the Reduced Professional Efficacy dimension was reverse-scored, with lower scores indicating more severe job burnout. A score of 3 on each dimension was used as the cutoff value to determine the presence of burnout; the scores of each dimension were independent and not summed. The presence of burnout in any one dimension was considered as having job burnout: burnout in only one dimension was mild job burnout, burnout in two dimensions was moderate job burnout, and burnout in all three dimensions was severe job burnout (Arvidsson et al., 2016). In this study, the Cronbach’s α coefficient of the total scale was 0.91, and the Cronbach’s α coefficients of the Emotional Exhaustion, Cynicism, and Reduced Professional Efficacy subscales were 0.93, 0.91, and 0.90, respectively.

Work Engagement: The Work Engagement Scale was derived from the UWES–9 version designed by Schaufeli et al. (2006), consisting of 9 items. The scale includes three dimensions: Vitality, Dedication, and Absorption, with 3 items measuring each dimension. A 5-point Likert scale was adopted, ranging from 1 (“very inconsistent”) to 5 (“very consistent”). The score of each subscale was the sum of the scores of the specific subscale. In this study, the Cronbach’s α coefficient of the total scale was 0.94, and the Cronbach’s α coefficients of the Vitality, Dedication, and Absorption subscales were 0.83, 0.86, and 0.88, respectively.

Work Connectivity Behavior After-hours: The scale for measuring WCBA compiled by Ma et al. (2016) based on the Chinese context was used. The instructions for the scale were as follows: “In your non-working hours over the past few weeks, how often have you used communication tools to handle work-related matters? Non-working hours include before work, lunch breaks, after work on weekdays, weekends, legal holidays, etc. Communication tools include mobile phones, computers, iPads, etc.; WeChat, QQ, email, etc. Please select the description that best fits your actual situation according to your perception and evaluation of yourself.” The scale includes 3 items: “The frequency with which I contact relevant personnel through the aforementioned communication tools for work matters (The frequency with which I take the initiative to contact others)”, “The frequency with which relevant personnel contact me through the aforementioned communication tools for work matters (the frequency with which I passively contact others)”, and “The frequency with which I check various work–related information (such as group messages, news, emails, online notifications, etc.)”. A 5-point Likert scale was adopted, where 1 indicated “Never”, 2 indicated “Seldom”, 3 indicated “Sometimes”, 4 indicated “Frequently”, and 5 indicated “Very Frequently”. The Cronbach’s α coefficient of the scale was 0.88.

### 2.3. Statistical Analysis

#### 2.3.1. Network Construction

This study used the bootnet package in R for psychological network estimation (Epskamp & Fried, 2018) and constructed a sparse partial correlation network based on the Gaussian Graphical Model (GGM). To reduce the influence of spurious correlations, we applied Graphical Least Absolute Shrinkage and Selection Operator (GLASSO) to regularize the covariance matrix of the variables, and selected the optimal regularization parameter (γ = 0.5) using the Extended Bayesian Information Criterion (EBIC), balancing model fitting and sparsity.

In the network model, nodes represent the psychological variables (including WFC, job burnout, work engagement, and WCBA), and edges represent the partial correlation between two nodes after controlling for other variables. Positive edges are depicted in blue, while negative edges are depicted in red. The thickness of the edges reflects the strength of the correlation.

The visualization of the network used the Fruchterman–Reingold spring algorithm (Fruchterman & Reingold, 1991) implemented via the qgraph package, a specialized tool for network visualization in psychometric research (Epskamp et al., 2012). This algorithm simulates a physical spring system, where strongly connected nodes are drawn to the center, and weakly connected nodes are pushed to the periphery, making it easier to identify the core variables and structural characteristics of the network. To assess the accuracy of the estimated edge weights, we applied a nonparametric bootstrap to calculate 95% confidence intervals (CIs) for each edge. If an edge’s original weight lies largely within its bootstrapped CI and the interval is narrow, it indicates high confidence in the stability of that edge’s estimate. We opted for a nonparametric bootstrap approach because parametric bootstrapping requires distributional assumptions that are difficult to verify and can be biased when applied to regularized networks like those estimated via EBICglasso. In contrast, the nonparametric bootstrap is fully data-driven and does not rely on such assumptions, making it the recommended choice for evaluating network accuracy under these conditions (Epskamp et al., 2018).

#### 2.3.2. Network Estimation

The accuracy and stability of the network structure and key node indicators were estimated and evaluated based on the standard process proposed by Epskamp et al. (2018). The estimation and evaluation involved two main aspects: network accuracy and stability analysis and node feature estimation

To assess the accuracy of the edge weight estimates, we used the nonparametric bootstrap method to calculate the 95% confidence intervals (CIs) for edge weights. If the original edge weight values largely fall within the confidence intervals and the intervals are narrow, it suggests high confidence in the edge weight estimates. Furthermore, we used the case-dropping bootstrap method to evaluate the stability of centrality indices, calculating the centrality stability (CS) coefficient. This coefficient measures the consistency of centrality indicators across different subsamples. According to Epskamp et al. (2018), the CS coefficient should be no less than 0.25; if it is higher than 0.50, it indicates that the centrality indicators have good stability.

To further analyze the structural position of each variable in the network, we calculated the following node indices:

Predictability: The predictability of each node was estimated using the mgm package (Haslbeck & Waldorp, 2018), which is the proportion of the variance of the node that can be explained by its directly adjacent nodes. This indicator can reflect the interdependence between variables in the network and the controllability of the system.

Expected Influence (EI): The algebraic sum of edge weights between a node and all its adjacent nodes was calculated using the qgraph package, which measures the overall influence of the node on other variables in the network. A higher expected influence value indicates a wider influence range of the node in the system.

Bridge Expected Influence (BEI): According to the definition proposed by Jones et al. (2021), BEI measures the connection strength of a node across multiple psychological constructs (such as job burnout and work engagement). A higher value indicates that the node has a more significant connecting role between different psychological modules and may be a key bridge in the process of symptom transmission and system integration.

## 3. Results

### 3.1. Accuracy and Stability of Network Estimation

**Figure 1:**
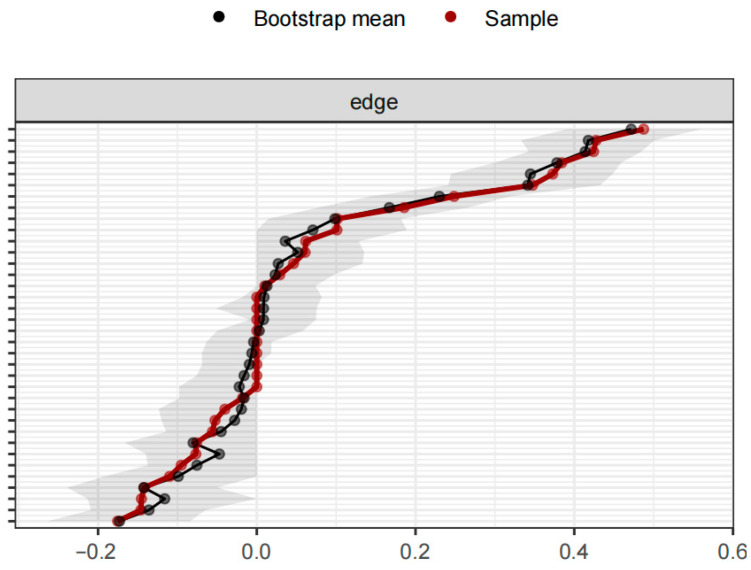
Accuracy of Edge Weight Estimation.

First, to assess the accuracy of the network edge weight estimation, we evaluated the overlap between the 95% confidence intervals (CIs) of the edge weights from the original data and the bootstrap resampled data. The results showed a high overlap, indicating a high level of confidence in the network edge weight estimates (Figure 1). Furthermore, the CS coefficient was calculated using the case-dropping bootstrap method to evaluate the stability of the network structure. The result showed that CS = 0.75, which is higher than the recommended threshold of 0.50, indicating that the network structure has a high level of stability (Epskamp et al., 2018).

### 3.2. Network Connectivity

Using the Gaussian Graphical Model (GGM), a network was constructed to examine the impact of WFC on the work-related psychological states (job burnout and work engagement) and behavioral work outcome (work connectivity behavior after hours) of university teachers. The network analysis revealed that, of the 36 possible edges, 27 (75.0%) were non-zero, indicating that the network exhibits a certain degree of sparsity.

**Figure 2:**
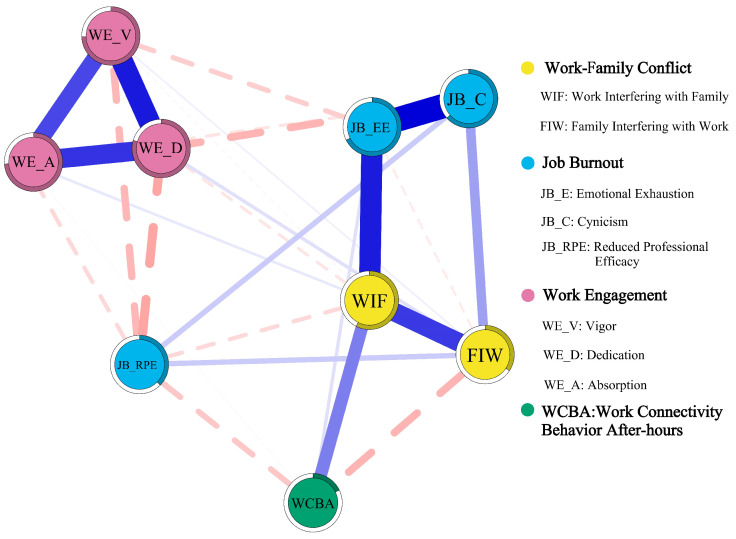
Partial correlation network among WFC, job burnout, work engagement, and WCBA. Note: The thickness of the edges indicates the degree of partial correlation between nodes. Positive (negative) correlations are represented by blue (red) edges. The color region around the nodes indicates the predictability of the node.

In this network, the nodes directly related to WIF include FIW (weight = 0.373), Emotional Exhaustion (weight = 0.424), WCBA (weight = 0.249), and Reduced Professional Efficacy (weight = −0.077). The nodes directly related to FIW include Cynicism (weight = 0.186), WCBA (weight = −0.145), Reduced Professional Efficacy (weight = 0.101), and Dedication (weight = 0.062). (Figure 2) As shown in Table 3, the strongest positive partial correlation across the entire network was observed between Emotional Exhaustion and Cynicism (weight = 0.487), which underscores the inherent and robust link between the two core dimensions of job burnout, even when the impacts of WFC, work engagement and WCBA are excluded. For the two bidirectional dimensions of WFC, WIF and FIW show a moderate positive partial correlation (weight = 0.373), demonstrating a certain degree of covariation between work-to-family and family-to-work conflict while also reflecting their relative structural independence, which aligns with the theoretical definition of bidirectional WFC as two distinct constructs. Additionally, the three dimensions of work engagement exhibit prominent internal positive correlations in Table 3: Vigor and Dedication (weight = 0.427), Dedication and Absorption (weight = 0.384), and Vigor and Absorption (weight = 0.348), all of which are moderate to strong correlations, indicating the high structural consistency and mutual reinforcement of work engagement in the network. Although Vigor is not directly associated with WIF, it is indirectly related through Emotional Exhaustion. Similarly, although Absorption and Emotional Exhaustion are not directly related to FIW, they are indirectly connected through WIF and FIW.

### 3.3. Predictability, Centrality, and Bridge Centrality of Network Nodes

**Figure 3:**
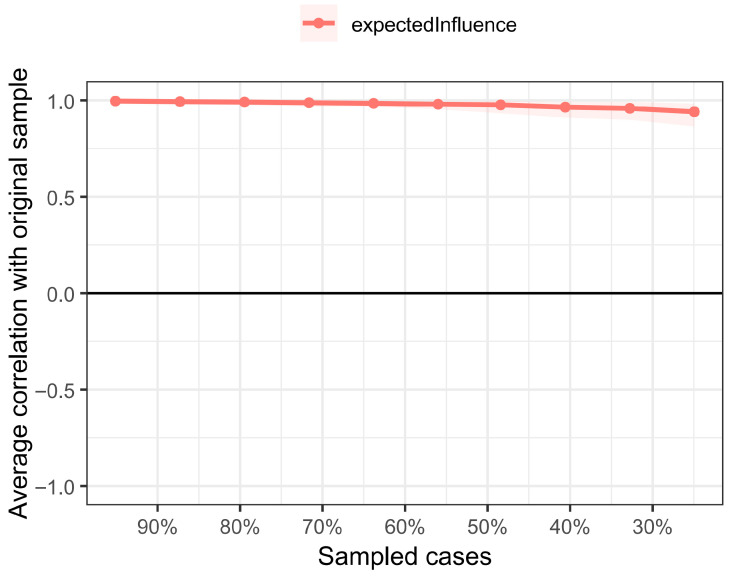
Bootstrap Results of Network Subsets. Note: Accuracy estimations of all edge weights using the non-parametric bootstrapping method (*N* = 409). Narrower CIs indicate reliable accuracy.

The CS coefficient indicates that Expected Influence (CS(cor = 0.7) = 0.751), it exceeds the critical value of 0.5 required in simulation studies, indicating that this indicator is stable (Figure 3). Therefore, the node Expected Influence is interpretable. The predictability analysis of the nodes showed (Figure 2, Table 4) that the predictability for WIF was 0.575 and for FIW it was 0.343, indicating that the connected nodes explained 57.5% of the variance in WIF and 34.3% in FIW. The nodes with the highest predictability in the network were the dimensions of work engagement: Vigor, Dedication, and Absorption.

**Figure 4:**
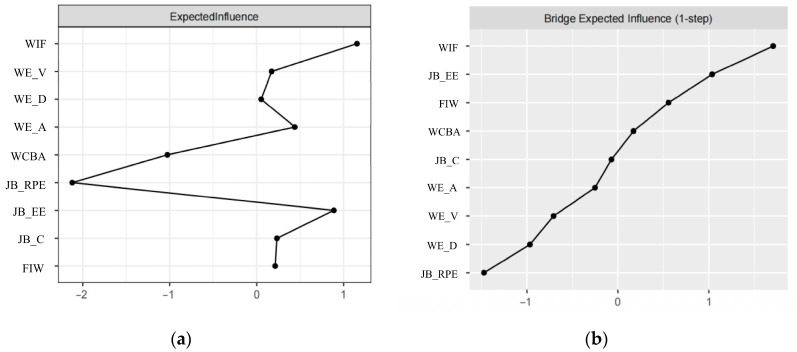
Centrality indices of the network. (**a**) Expected Influence; (**b**) Bridge Expected Influence. Note: WE_V = Vigor; WE_D = Dedication; WE_A = Absorption; JB_RPE = Reduced Professional Efficacy; JB_EE = Emotional Exhaustion; JB_C = Cynicism.

Centrality analysis showed (Figure 4a, Table 4) that the WIF node had the highest expected influence (EI = 1.154), indicating it is the most influential node in the network, holding a central position. This suggests that WIF is the most influential factor affecting other nodes in the system. The Bridge Expected Influence (BEI) reflects the node’s ability to connect different psychological constructs within the network. WIF also exhibits the highest BEI value of 0.572, indicating its critical bridging role. It connects multiple dimensions, such as Job Burnout (particularly Emotional Exhaustion) and Work Engagement (especially Absorption), thus playing a key role in transmitting effects between these domains. This highlights the pivotal function of WIF in the psychological system, facilitating the transmission of work-related stress into emotional exhaustion and work connectivity behavior. The Predictability analysis reveals that WIF has a predictability of 0.575, suggesting that it significantly influences the connected variables in the network. Work engagement dimensions, including Vigor, Dedication, and Absorption, show high predictability, meaning these variables are especially sensitive to changes in the network, particularly due to work-family conflict. Finally, the high predictability of work engagement dimensions emphasizes their importance in buffering against burnout and maintaining professional well-being.

The results of bridge expected influence centrality showed that WIF had the highest bridge expected influence value. WIF is a bridging node connecting the dimensions of job burnout and work engagement, FIW, and WCBA (Figure 4b, Table 4). Specifically, FIW in WFC (weight = 0.373), Emotional Exhaustion in job burnout (weight = 0.424), and Absorption in work engagement (weight = 0.046) are respectively associated with the WIF dimension.

## 4. Discussion

This study aimed to explore the impact of WFC on the work-related psychological states (job burnout and work engagement) and behavioral work outcome (work connectivity behavior after hours) of university teachers, focusing on the complex network relationships between job burnout, work engagement, and WCBA. We adopted Psychological Network Analysis to analyze data from 409 university teachers, constructing a partial correlation network model to reveal the systemic influence paths of WFC. The results show WIF as the core hub in the network—with the highest centrality and bridging effect—directly linking emotional exhaustion, indirectly weakening work engagement via reduced professional efficacy, and forming a dynamic feedback loop with WCBA.

### 4.1. The Pivotal Role of WFC in the Network

The analysis demonstrated that job burnout, work engagement, and work connectivity behavior after-hours are interconnected with WFC within a complex network structure. Emotional exhaustion, cynicism, reduced professional efficacy, vigor, dedication, and absorption are indeed interrelated in the network. The study found that WIF has the highest Expected Influence and Bridge Expected Influence—conceptually, Expected Influence measures a node’s overall centrality by summing the weighted values (including positive and negative) of its direct connections with all other nodes, reflecting the total impact of the node on the entire network (Robinaugh et al., 2016); while Bridge Expected Influence specifically identifies bridge nodes that link different psychological clusters (e.g., job burnout, work engagement), highlighting their cross-dimensional connecting function (Jones et al., 2021). This dual high centrality indicates that WIF not only directly affects various psychological states but also serves as a key bridge connecting multiple dimensions in the psychological system.

WFC occupies a central role, deeply influencing various sub-dimensions such as job burnout, work engagement, and WCBA. This finding is consistent with the core tenet of the Conservation of Resources Theory (Hobfoll, 1989), which posits that resource threat or loss triggers a chain reaction of psychological depletion and adaptive behaviors (Demerouti, 2025). Specifically, university teachers constantly facing work-related disruptions to their family life face reduced family support, obstructed recovery activities, and blurred boundaries, which in turn triggers a series of depletion responses within the psychological system. This study, through network analysis, further elucidates that WIF is not only a starting factor or predictor but also a key node in the psychological system, with its bridging function providing a more systematic perspective for intervention strategies. This finding supports focusing on WIF as a priority in interventions aimed at improving teachers’ mental health, helping to develop more targeted boundary support, flexible working systems, and teacher empowerment policies to alleviate its systemic psychological impacts.

### 4.2. The Impact of WIF on Emotional Exhaustion

The results showed that WIF is strongly associated with emotional exhaustion, which is one of the most tightly connected links in the network. This finding suggests that teaching as a profession inherently involves high emotional labor and responsibility (Chen et al., 2025). When work roles frequently invade family spaces, individuals continuously deplete personal resources in the long run, leading to emotional exhaustion and fatigue. Eventually, this manifests as emotional exhaustion, cynicism, and reduced professional efficacy, typical burnout responses (Yang & Chen, 2022). This process can be explained through the Conservation of Resources theory, where teachers, in their efforts to balance teaching responsibilities with family obligations, experience dual resource depletion pressure. Once resources are not effectively replenished, burnout responses are triggered (Burić et al., 2023). This indicates that interventions targeting work interference should not only focus on reducing the frequency of conflicts but also on improving teachers’ resource adjustment abilities and recovery opportunities. For example, setting clear work-time boundaries, providing family support resources, and establishing psychological adjustment mechanisms could help sever the root causes of burnout (Dodanwala & Shrestha, 2021).

WIF’s negative relationship with Reduced Professional Efficacy warrants additional discussion. Although one might expect WIF to directly reduce professional efficacy, the results show a more complex dynamic: teachers experiencing WIF may temporarily overcompensate by exerting greater effort in their professional duties to maintain their perceived competence, despite emotional depletion. This overcompensation may be driven by an underlying fear of failing to meet professional expectations or due to the anxiety induced by WIF, where teachers strive to demonstrate their continued efficacy and dedication (Nayak et al., 2025). While this overcompensation may provide short-term boosts in professional efficacy, it is ultimately unsustainable and leads to greater emotional exhaustion over time. As a result, the professional efficacy that WIF influences is weakened indirectly, as the continued depletion of emotional resources exacerbates burnout and impairs long-term work engagement (Tian et al., 2024).

### 4.3. The Impact of WIF on Work Engagement

This study found that the direct effect of FIW on work engagement was relatively weak, but it did show that FIW indirectly affects work engagement through its mediating role in reduced professional efficacy. This pattern indicates that after teachers experience work–family resource conflict, they first experience a significant decrease in their sense of personal efficacy, feeling unable to complete tasks efficiently or meet students’ demands. This results in reduced active engagement in work. Particularly, the dimensions of dedication and vigor were highly predictable in the network analysis, indicating that these positive psychological states are easily influenced by fluctuations in other variables in the network and are most difficult to maintain in highly conflicted environments. This phenomenon can be explained using the psychological capital theory, suggesting that teachers’ work engagement depends not only on external resources but also on self-efficacy regulation (Xiong & Ye, 2014). When individuals are in a prolonged state of resource depletion, their reserve of positive psychological resources gradually depletes, leading to a significant decline in engagement (Y. Li, 2019). Notably, organizational work–family culture also plays a pivotal role in this process–work–family culture can indirectly affect work engagement by regulating the intensity of WFC (Bian & Qian, 2019), which provides additional insights for organizational intervention.

### 4.4. The Relationship Between WIF and WCBA

The study revealed that WCBA is significantly positively correlated with WIF and is closely associated with emotional exhaustion. This suggests that WCBA may amplify WFC through emotional exhaustion. This result indicates that when university teachers feel burdened by emotional resource depletion or unfinished work tasks, they tend to continue handling teaching matters or maintain work communication connectivity even during non-work hours. On the surface, this may appear as proactive behavior driven by a sense of responsibility, but in reality, this technological connectivity likely reflects a lack of boundary management between work and family (Harris et al., 2022). This is consistent with prior research indicating that if teachers lack sufficient time to disengage after work, it can exacerbate their emotional exhaustion and cognitive fatigue, creating a vicious cycle of “the more exhausted, the more they work, and the more they work, the more exhausted they become” (Park & Shin, 2020).

Additionally, WIF-related anxiety might also contribute to the increase in WCBA. Teachers experiencing high levels of WIF may feel overwhelmed by the perception that they are failing to meet work demands, leading to anxiety and a sense of responsibility that spills over into their personal time. This anxiety can drive them to engage in WCBA as a coping mechanism to manage work-related tasks or concerns that remain unresolved, even after work hours (Nayak et al., 2025). As a result, while WCBA may appear to be a proactive form of engagement, it may reflect an underlying struggle to balance work and family life, driven by anxiety or organizational pressure.

Information and communication technology (ICT) has a dual role—it helps teachers gain work flexibility but simultaneously exposes them to continuous emotional depletion due to sustained connectivity. For instance, the remote work facilitated by ICT has been shown to induce technological stress, negatively impacting mental health and increasing work–life conflict. Moreover, communication via ICT during non-work hours has also been found to positively influence job burnout (Karimikia et al., 2021). It highlights the need for both organizational interventions, such as clarifying expectations for after-hours work, and individual strategies to manage stress and anxiety. By addressing these factors, universities can support teachers in maintaining healthier boundaries between work and family life, thereby reducing the detrimental impact of WCBA on teachers’ psychological well-being.

### 4.5. Limitations and Future Directions

Despite the contributions of this study, there are several limitations. First, the study used a cross-sectional design, which limits causal inferences. The relationships between WFC, job burnout, work engagement, and WCBA should be further explored using longitudinal designs to understand the dynamic evolution of these variables over time. Second, while the network model reveals complex relationships between the variables, potential moderating factors such as gender roles, family structure, and cultural context have not been fully explored. Future research could incorporate multi-level analysis or mixed methods to deepen the understanding of these moderators and their effects on the psychological system. Moreover, it is important to recognize some critiques of network analysis in psychological research. Limitations in applying network analysis to psychometrically based studies, particularly regarding the assumptions of linearity and the complexity of psychological networks, have been highlighted (Neal et al., 2022). The boundary specification problem for centrality in psychological networks also suggests that more attention should be given to the ways in which centrality measures are defined and interpreted in such models (Neal & Neal, 2023). These critiques emphasize the need for careful consideration of the methods used in network analysis to avoid misinterpretations of the relationships between variables. Lastly, this study focused on university teachers, but subgroups within the teaching population, such as counselors, special education teachers, and administrative staff, may have different experiences of WFC and coping strategies. Future studies could compare these subgroups to better understand their unique challenges and needs in the context of WFC.

With the help of network analysis, we have successfully identified the nodes and paths most valuable for intervention, thereby providing more precise and structured guidance for promoting teachers’ mental health. In the future, universities can further optimize institutional arrangements and organizational support systems from three key dimensions: boundary management, resource allocation, and information communication management, with a view to reducing the psychological burden caused by conflicts and improving teachers’ occupational well-being and work performance. In addition, subsequent research can consider cross-group comparisons or introduce a cultural comparison perspective to explore the moderating role of cultural backgrounds on the psychological mechanisms of WFC. With the deep integration of artificial intelligence and information technology, teachers’ work models are undergoing profound changes. In the future, there is an urgent need to strengthen research on how ICT penetrates and affects new roles and risk mechanisms in the WFC network. Cross-lagged panel networks reveal item-level longitudinal effects within and across constructs over time (Wysocki et al., 2022). Unlike undirected networks typically estimated from cross-sectional data, paths in Cross-lagged panel networks are directed from measurement time t to a later measurement time t + 1. Future research may adopt Cross-lagged panel networks to conduct systematic causal analyses of the psychological characteristics of university teachers, explore the specific psychological mechanisms underlying work-family conflict among university teachers, and provide more convincing evidence and recommendations for mental health interventions targeting this population.

### 4.6. Implications for Theory and Practice

This study extends the application of COR theory to WFC research by revealing the network associations between WFC, work-related psychological states, and behavioral work outcome. We confirm the unique role of WIF as a core hub in the psychological system, supplementing the existing literature on the dimensional differences in WFC (Casper et al., 2007; Greenhaus & Beutell, 1985). We integrated WCBA into the network model, enriching the understanding of work–family interaction mechanisms in the digital era.

For universities and educational institutions, the findings suggest prioritizing interventions targeting WIF. Specific measures include establishing clear work-time boundaries, providing flexible work arrangements, and offering family support resources. For individual teachers, our results highlight the importance of boundary management and resource recovery—reducing unnecessary WCBA to break the vicious cycle of resource depletion. Additionally, training programs on psychological capital enhancement can help mitigate the indirect impact of WIF on work engagement (Lyu & Fan, 2022).

## 5. Conclusions

This study systematically reveals, through network analysis, the complex mechanisms by which WFC influences university teachers’ work-related psychological states and behavioral work outcome. The results indicate that WIF functions as the core hub and bridging node within the psychological system, showing direct and close connections with emotional exhaustion, work engagement, and WCBA. These findings suggest that WIF can be considered a central intervention target, which is of critical importance for maintaining teachers’ occupational mental health. Intervention strategies that strengthen work–life boundary management and appropriately regulate work role demands may effectively alleviate WFC and its associated negative consequences.

## Figures and Tables

**Table 1:** Demographic Characteristics of Participants (*N* = 409).

Variable	Group	Frequency (f)	Percentage (%)
Gender			
	Male	179	43.8
	Female	230	56.2
Age			
	25 years and below	3	0.7
	25–30 years	50	12.2
	31–35 years	99	24.2
	36–40 years	93	22.7
	41–45 years	81	19.8
	46–50 years	47	11.5
	51 years and above	36	8.8

**Table 2:** Descriptive statistics and reliability for the study variables.

Factor	Mean (SD)	Variance	Skew	Kurtosis	α
Work–Family Conflict					
Work Interfering with Family	30.503 (8.0431)	64.692	−0.318	−0.478	0.915
Family Interfering with Work	23.826 (7.7229)	59.643	0.286	−0.250	0.913
Job Burnout					
Emotional Exhaustion	15.090 (4.568)	20.867	0.129	−0.242	0.930
Cynicism	9.828 (3.9294)	15.440	0.489	−0.259	0.911
Reduced Professional Efficacy	12.020 (4.221)	17.821	1.098	2.361	0.902
Work Engagement					
Vigor	10.150 (2.446)	5.982	−0.155	0.029	0.828
Dedication	10.670 (2.425)	5.882	−0.324	0.256	0.863
Absorption	10.150 (2.594)	6.729	−0.246	0.138	0.876
Work Connectivity Behavior After-hours					
Work Connectivity Behavior After-hours	12.510 (2.175)	4.731	−0.656	0.129	0.881

**Table 3:** Network Partial Correlation Weight Coefficient.

	1	2	3	4	5	6	7	8	9
Work Interfering with Family	–								
Family Interfering with Work	0.373	–							
Emotional Exhaustion	0.424	–0.04	–						
Cynicism	0	0.186	0.487	–					
Reduced Professional Efficacy	–0.077	0.101	0	0.101	–				
Vigor	–0.018	0.029	–0.095	0	–0.142	–			
Dedication	–0.053	0.062	0	–0.146	–0.175	0.427	–		
Absorption	0.046	0	0	–0.056	–0.076	0.348	0.384	–	
Work Connectivity Behavior After-hours	0.249	–0.145	0.061	0	–0.11	0	0	0.01	–

**Table 4:** Predictability and Centrality Indices of Network Nodes.

Node	Predictability	Expected Influence	Bridge Expected Influence
Work–Family Conflict	–	–	–
Work Interfering with Family	0.575	1.154	0.572
Family Interfering with Work	0.343	0.212	0.192
Job Burnout	–	–	–
Emotional Exhaustion	0.676	0.888	0.350
Cynicism	0.626	0.232	−0.016
Reduced Professional Efficacy	0.374	−2.122	−0.479
Work Engagement	–	–	–
Vigor	0.746	0.173	−0.226
Dedication	0.795	0.051	−0.312
Absorption	0.739	0.439	−0.076
Work Connectivity Behavior After-hours	0.177	−1.027	0.064

## Data Availability

The data presented in this study are available on request from the corresponding author.

## Abbreviations

The following abbreviations are used in this manuscript:

WFC	Work–Family Conflict
WIF	Work Interfering with Family
FIW	Family Interfering with Work
WCBA	Work Connectivity Behavior After-hours
ICT	Information and Communication Technology

## References

[B1-behavsci-16-00322] Agarwal U. A., Baral R., Rastogi M. (2025). Work–family conflict and work engagement among construction professionals: Role of psychological contract breach and gender. Engineering, Construction and Architectural Management.

[B2-behavsci-16-00322] Anjum M. A., Liang D., Durrani D. K., Parvez A. (2022). Workplace mistreatment and emotional exhaustion: The interaction effects of self–compassion. Current Psychology.

[B3-behavsci-16-00322] Arvidsson I., Håkansson C., Karlson B., Björk J., Persson R. (2016). Burnout among Swedish school teachers—A cross–sectional analysis. BMC Public Health.

[B4-behavsci-16-00322] Bakker A. B., De Vries J. D. (2021). Job Demands–Resources theory and self–regulation: New explanations and remedies for job burnout. Anxiety, Stress, & Coping.

[B5-behavsci-16-00322] Balogun A. G., Afolabi O. A. (2019). Examining the moderating roles of job demands and resources on the relation between work engagement and work–family conflict. South African Journal of Psychology.

[B6-behavsci-16-00322] Bao X., Dong J., Guo J. (2025). Family-to-work conflict and innovative work behavior among university teachers: The mediating effect of work stress and the moderating effect of gender. Behavioral Sciences.

[B7-behavsci-16-00322] Bian J. L., Qian Y. Y. (2019). The impact of work–family culture on employees’ work engagement: The mediating role of work–family conflict. Enterprise Economy.

[B8-behavsci-16-00322] Burić I., Šimunović M., Balaž B. (2023). Work and family conflicts and teacher commitment during the COVID–19 pandemic: A moderated mediation analysis of emotional exhaustion and psychological capital. Educational Psychology.

[B9-behavsci-16-00322] Capone V., Petrillo G. (2020). Mental health in teachers: Relationships with job satisfaction, efficacy beliefs, burnout and depression. Current Psychology.

[B10-behavsci-16-00322] Carlson D. S., Kacmar K. M., Williams L. J. (2000). Construction and initial validation of a multidimensional measure of work–family conflict. Journal of Vocational Behavior.

[B11-behavsci-16-00322] Casper W. J., Eby L. T., Bordeaux C., Lockwood A., Lambert D. (2007). A review of research methods in IO/OB work–family research. Journal of Applied Psychology.

[B12-behavsci-16-00322] Chen Y., Sun S., Liu X. (2025). The relationship between teacher emotional labor and work/family conflict: The mediating role of teacher–colleague relations. Acta Psychologica.

[B13-behavsci-16-00322] Demerouti E. (2025). Job demands–resources and conservation of resources theories: How do they help to explain employee well–being and future job design?. Journal of Business Research.

[B14-behavsci-16-00322] Dodanwala T. C., Shrestha P. (2021). Work–family conflict and job satisfaction among construction professionals: The mediating role of emotional exhaustion. On the Horizon: The International Journal of Learning Futures.

[B15-behavsci-16-00322] Epskamp S., Borsboom D., Fried E. I. (2018). Estimating psychological networks and their accuracy: A tutorial paper. Behavior Research Methods.

[B16-behavsci-16-00322] Epskamp S., Cramer A. O., Waldorp L. J., Schmittmann V. D., Borsboom D. (2012). qgraph: Network visualizations of relationships in psychometric data. Journal of Statistical Software.

[B17-behavsci-16-00322] Epskamp S., Fried E. I. (2018). A tutorial on regularized partial correlation networks. Psychological Methods.

[B18-behavsci-16-00322] Fiorilli C., Buonomo I., Romano L., Passiatore Y., Iezzi D. F., Santoro P. E., Benevene P., Pepe A. (2020). Teacher confidence in professional training: The predictive roles of engagement and burnout. Sustainability.

[B19-behavsci-16-00322] Fruchterman T. M., Reingold E. M. (1991). Graph drawing by force–directed placement. Software: Practice and Experience.

[B20-behavsci-16-00322] Geraldes D. T., Chambel M. J., Carvalho V. S. (2025). Work–family practices and work–family relationship: The role of boundary management. BMC Public Health.

[B21-behavsci-16-00322] Greenhaus J. H., Beutell N. J. (1985). Sources of conflict between work and family roles. Academy of Management Review.

[B22-behavsci-16-00322] Harris K. J., Harris R. B., Valle M., Carlson J., Carlson D. S., Zivnuska S., Wiley B. (2022). Technostress and the entitled employee: Impacts on work and family. Information Technology & People.

[B23-behavsci-16-00322] Haslbeck J. M., Waldorp L. J. (2018). How well do network models predict observations? On the importance of predictability in network models. Behavior Research Methods.

[B24-behavsci-16-00322] Hetrick A. L., Haynes N. J., Clark M. A., Sanders K. N. (2024). The theoretical and empirical utility of dimension–based work–family conflict: A meta–analysis. Journal of Applied Psychology.

[B25-behavsci-16-00322] Hobfoll S. E. (1989). Conservation of resources: A new attempt at conceptualizing stress. American Psychologist.

[B26-behavsci-16-00322] Hobfoll S. E. (2001). The influence of culture, community, and the nested–self in the stress process: Advancing conservation of resources theory. Applied Psychology.

[B27-behavsci-16-00322] Johnston K., Corbett S., Bezuidenhout A., van Zyl D., Pasamar S. (2023). Gender differences in work-life conflict during Covid? A research agenda for work-life conflict post-pandemic. Research in Post-Compulsory Education.

[B28-behavsci-16-00322] Jones P. J., Ma R., McNally R. J. (2021). Bridge centrality: A network approach to understanding comorbidity. Multivariate Behavioral Research.

[B29-behavsci-16-00322] Karimikia H., Singh H., Joseph D. (2021). Negative outcomes of ICT use at work: Meta–analytic evidence and the role of job autonomy. Internet Research.

[B30-behavsci-16-00322] Koura U., Sekine M., Yamada M., Tatsuse T. (2020). The health effects of work-family conflict in men and women Japanese civil servants: A longitudinal study. Industrial Health.

[B31-behavsci-16-00322] Li C. P., Shi K. (2003). The impact of distributive justice and procedural justice on job burnout. Acta Psychologica Sinica.

[B32-behavsci-16-00322] Li M. J., Wang Z. H., Liu Y. (2015). Work family conflicts and job burnout in primary and middle school teachers: The mediator role of self–determination motivation. Psychological Development and Education.

[B33-behavsci-16-00322] Li Y. (2019). Leadership styles and knowledge workers’ work engagement: Psychological capital as a mediator. Current Psychology.

[B34-behavsci-16-00322] Listau K., Christensen M., Innstrand S. T. (2017). Work engagement: A double–edged sword? A study of the relationship between work engagement and the work–home interaction using the ARK research platform. Scandinavian Journal of Work and Organizational Psychology.

[B35-behavsci-16-00322] Lyu X., Fan Y. (2022). Research on the relationship of work family conflict, work engagement and job crafting: A gender perspective. Current Psychology.

[B36-behavsci-16-00322] Ma H., Xie J., Tang H., Shen C., Zhang X. (2016). Relationship between working through information and communication technologies after hours and well–being among Chinese dual–earner couples: A spillover–crossover perspective. Acta Psychologica Sinica.

[B37-behavsci-16-00322] Maslach C., Schaufeli W. B., Leiter M. P. (2001). Job burnout. Annual Review of Psychology.

[B38-behavsci-16-00322] Mody L., Griffith K. A., Jones R. D., Stewart A., Ubel P. A., Jagsi R. (2022). Gender differences in work-family conflict experiences of faculty in academic medicine. Journal of General Internal Medicine.

[B39-behavsci-16-00322] Nayak S., Budhwar P., Malik A. (2025). Technostress of HR professionals: The darker implication of remote work transformations. The International Journal of Human Resource Management.

[B40-behavsci-16-00322] Neal Z. P., Forbes M. K., Neal J. W., Brusco M. J., Krueger R., Markon K., Steinley D., Wasserman S., Wright A. G. (2022). Critiques of network analysis of multivariate data in psychological science. Nature Reviews Methods Primers.

[B41-behavsci-16-00322] Neal Z. P., Neal J. W. (2023). Out of bounds? The boundary specification problem for centrality in psychological networks. Psychological Methods.

[B42-behavsci-16-00322] Park E. Y., Shin M. (2020). A meta–analysis of special education teachers’ burnout. Sage Open.

[B43-behavsci-16-00322] Purwayoga P. V. S., Dharmanegara I. B. A., Yasa P. N. S. (2019). Mediating role of work engagement and emotional exhaustion in the effect of work–family conflict on female workers’ turnover intention. International Journal of Academic Research in Business and Social Sciences.

[B44-behavsci-16-00322] Raja U., Javed Y., Abbas M. (2018). A time lagged study of burnout as a mediator in the relationship between workplace bullying and work–family conflict. International Journal of Stress Management.

[B45-behavsci-16-00322] Robinaugh D. J., Millner A. J., McNally R. J. (2016). Identifying highly influential nodes in the complicated grief network. Journal of Abnormal Psychology.

[B46-behavsci-16-00322] Rubio C., Osca A., Recio P., Urien B., Peiró J. M. (2015). Work–family conflict, self–efficacy, and emotional exhaustion: A test of longitudinal effects. Revista de Psicología del Trabajo y de las Organizaciones.

[B47-behavsci-16-00322] Schaufeli W. B., Bakker A. B., Salanova M. (2006). The measurement of work engagement with a short questionnaire: A cross–national study. Educational and Psychological Measurement.

[B48-behavsci-16-00322] Schaufeli W. B., Salanova M., González–Romá V., Bakker A. B. (2002). The measurement of engagement and burnout: A two sample confirmatory factor analytic approach. Journal of Happiness Studies.

[B49-behavsci-16-00322] Shao Y., Jiang W., Zhu H., Zhang C., Xu W. (2025). The relationship between work stress and well–being among Chinese primary and secondary school teachers: The chain mediation of affective rumination and work engagement. BMC Psychology.

[B50-behavsci-16-00322] Skinner N., Pocock B. (2008). Work–life conflict: Is work time or work overload more important?. Asia Pacific Journal of Human Resources.

[B51-behavsci-16-00322] Song D., Zhao J., Wu H., Ji X. (2025). The impact of work–family conflict on job burnout among community social workers in China. PLoS ONE.

[B52-behavsci-16-00322] Su Q., Jiang M. (2023). “Ideal employees” and “good wives and mothers”: Influence mechanism of bi–directional work–family conflict on job satisfaction of female university teachers in China. Frontiers in Psychology.

[B53-behavsci-16-00322] Tian M., Li X., Ma J., Zhang T., Wang P., Yuan X., Wang X. (2024). Who merits more concern: University teachers under task–related or those under interpersonal–related stress?. Humanities and Social Sciences Communications.

[B54-behavsci-16-00322] Usset T. J., Baker L. D., Griffin B. J., Harris J. I., Shearer R. D., Munson J., Godzik C., Torrey W. C., Bardach S. H., Mulley A. G., Locke A., Wright H. M., Call M., Sexton B., Shanafelt T., Smith A. J. (2024). Burnout and turnover risks for healthcare workers in the United States: Downstream effects from moral injury exposure. Scientific Reports.

[B55-behavsci-16-00322] Wang F., Li Y. (2023). Social media use for work during non–work hours and work engagement: Effects of work–family conflict and public service motivation. Government Information Quarterly.

[B56-behavsci-16-00322] Wu G., Wu Y., Li H., Dan C. (2018). Job burnout, work–family conflict and project performance for construction professionals: The moderating role of organizational support. International Journal of Environmental Research and Public Health.

[B57-behavsci-16-00322] Wysocki A., Rhemtulla M., Van Bork R., Cramer A. O. J. (2022). Cross-lagged network models. PsyArXiv.

[B58-behavsci-16-00322] Xiong M., Ye Y. D. (2014). The concept, measurement, influencing factors and effects of psychological capital. Journal of East China Normal University (Educational Sciences).

[B59-behavsci-16-00322] Yang C. J., Chen A. B. (2022). Work–family conflict, organizational identification, and professional identification among Chinese nurses from a resource perspective. Journal of Nursing Research.

[B60-behavsci-16-00322] Yuan F., Wang J. (2025). How does digital leadership affect teachers’ life satisfaction in primary and secondary schools: The sequential mediating role of digital technology skills and work engagement. Medicine.

[B61-behavsci-16-00322] Zhao J. (2023). Meta–analysis on the relationship between work–family conflict and job burnout of teachers. Journal of Yancheng Teachers University (Humanities & Social Sciences Edition).

